# P-1868. Incidence of Rhabdomyolysis in Patients Receiving Daptomycin in a Community Hospital OPAT Program

**DOI:** 10.1093/ofid/ofae631.2029

**Published:** 2025-01-29

**Authors:** Allison N Cid, Heather D Gibson, Gretchen S Arnoczy, Jaspaul S Jawanda, Madonna A Biritwum, Amanda Di-Majo, Anna LeViere

**Affiliations:** FirstHealth of the Carolinas Moore Regional Hospital, Pinehurst, North Carolina; FirstHealth of the Carolinas Moore Regional Hospital, Pinehurst, North Carolina; FirstHealth of the Carolinas, Pinehurst, North Carolina; n/a, Pinehurst, NC; FirstHealth, Southern Pines, North Carolina; FirstHealth of the Carolinas Moore Regional Hospital, Pinehurst, North Carolina; FirstHealth of the Carolinas Moore Regional Hospital, Pinehurst, North Carolina

## Abstract

**Background:**

Daptomycin is a favorable medication for outpatient parenteral antimicrobial therapy (OPAT) due to its low rate of serious adverse events, however one potential therapy complication is the development of rhabdomyolysis. The aim of this study was to determine the incidence of rhabdomyolysis within a community hospital’s OPAT program.

**Methods:**

A retrospective chart review was performed of patients enrolled in the OPAT program and who received daptomycin therapy from December 2022 to August 2023. The primary outcome of the study was the percentage of patients who developed rhabdomyolysis while on daptomycin therapy. Secondary outcomes included the determination of any patient- or drug-specific risk factors that may have increased the risk of rhabdomyolysis. Patients were included if they were discharged on daptomycin and followed by the formal OPAT program during the study period. Patients who were less than 18 years of age or pregnant were excluded.

**Results:**

There were 83 patients who received daptomycin during the study period and 5/83 (6%) developed rhabdomyolysis. There were 8 (9.6%) patients who had an elevated creatinine kinase (CK) but did not have further progression to rhabdomyolysis. The median daptomycin dose given to patients was 8 mg/kg. Four out of the five patients with rhabdomyolysis were female (80%). The mean BMI of those who developed rhabdomyolysis was 37.9. The mean onset of rhabdomyolysis was 10.8 days. No patients with rhabdomyolysis were on a concomitant statin. None of the five patients experienced death or permanent organ failure.

**Conclusion:**

The development of rhabdomyolysis in our study was higher than anticipated based on previous phase 3 studies. This difference may be due to higher dosing used between studies. Potential factors to consider for increased risk of rhabdomyolysis based on this study include female gender and BMI greater than 35. Statin use did not appear to be associated with an increased risk of rhabdomyolysis. Daptomycin is still a safe and effective antibiotic in the OPAT setting, however CK should be closely monitored.

**Disclosures:**

All Authors: No reported disclosures

